# Causes and risk factors of an unplanned second craniotomy in patients with traumatic brain injury

**DOI:** 10.1186/s12893-023-01977-w

**Published:** 2023-04-04

**Authors:** Shilong Fu, Pengwei Hou, Guofeng Wang, Shousen Wang

**Affiliations:** 1 Department of Neurosurgery, The First Hospital of Putian City, 351100 Putian, People’s Republic of China; 2 Department of Neurosurgery, 900 Hospital of The Joint Logistics Team, No. 156 Xihuanbei Road, Fuzhou, 350025 People’s Republic of China

**Keywords:** Traumatic brain injury, Unplanned second craniotomy, Risk factor, Cause analysis

## Abstract

**Background:**

The purpose of this retrospective study was to evaluate the causes and risk factors of an unplanned second craniotomy in patients with traumatic brain injury (TBI).

**Methods:**

A total of 219 patients with TBI who underwent initial unilateral intracranial supratentorial surgery between January 2016 to November 2021 were included. We evaluated the causes of an unplanned second craniotomy in 40 patients, and analyzed the risk factors for a contralateral second craniotomy in 21 patients using a multivariate logistic regression analysis.

**Results:**

The most common cause for an unplanned second craniotomy was delayed or enlarged hematoma in the non-operation area (26/40; 65%), followed by recurrent hematoma in the operation area (8/40; 20%), ipsilateral massive cerebral infarction (3/40; 7.5%), diffuse brain swelling (2/40; 5%) and enlarged cerebral contusion (1/40; 2.5%). Multivariate logistic regression analysis showed that a contralateral craniocerebral injury feature (CCIF) (OR = 13.175), defined on preoperative computerized tomography scanning, was independent risk factor for a contralateral second craniotomy.

**Conclusions:**

An unplanned second craniotomy in patients with TBI was mainly related to delayed or enlarged hematoma. An increased risk of a contralateral second craniotomy occurs in patients with CCIF.

## Background

Traumatic brain injury (TBI) is a global public health problem, which seriously threatens human life and health and has an average incidence of 235/100,000/year in European countries [1]. In addition, the overall mortality rates of patients with TBI and severe TBI are 4.8% and 19.7% in China, respectively [2]. Intracranial surgery, a procedure aimed at removing intracranial space-occupying hematoma and/or decompressive craniectomy (DC) to control intractable intracranial hypertension, has been proven to significantly increase survival rates in patients with severe TBI [1]. Previous case studies have reported that a catastrophic event occurs in patients with TBI after their initial unilateral intracranial surgery, such as a massive cerebral infarction secondary to extradural hematoma (EDH) evacuation [3, 4], a delayed EDH or subdural hematoma (SDH) following contralateral intracranial hematoma evacuation [5–8]. In such cases, an unplanned second craniotomy usually has to be performed immediately to save patient’s life, potentially leading to additional medical expenses and heavy family burdens. However, unplanned second craniotomies in patients with TBI are not well described in the literature. Therefore, The purpose of this retrospective study was to evaluate the causes and corresponding risk factors of an unplanned second craniotomy in patients with TBI, hoping to provide some useful experiences and lessons for neurosurgeons in treating patients with TBI.

## Methods

### Patient population

The clinical data of patients with TBI who underwent initial unilateral intracranial supratentorial surgery, treated in our institute, were collected from January 2016 to November 2021 and analysed retrospectively. All patients fulfilled the following criteria: (1) isolated TBI, without concomitant injuries, (2) patients whose initial surgery performed within 24 h after injury, (3) patients who required intracranial surgery once or twice after admission and (4) age ≥ 18 years old. Exclusion criteria were: (1) patients with the history of brain disease, craniocerebral trauma, and/or craniotomy, (2) patients with the history of oral anticoagulation or antiplatelet drugs, (3) patients without preoperative or postoperative computerized tomography (CT) image and (4) family members refused a second craniotomy. This study was approved by the Ethics Committee of The First Hospital of Putian City and complied with the Declaration of Helsinki.

### Clinical indices

In this study, we evaluated clinical indices including sex, age, basic diseases (hypertension or diabetes), Glasgow Coma Scale (GCS) score on admission, traumatic coagulopathy (defined as any of the following conditions based on reference values proposed by local institutions and laboratories: platelets < 125 × 10^9^/L, international normalized ratio > 1.2, and fibrinogen < 1.5 g/L), preoperative brain herniation (defined as unilateral or bilateral dilated pupils), contralateral craniocerebral injury feature (CCIF) defined on preoperative CT scanning [including initial hematoma not needing surgery (EDH, SDH and intracerebral hematoma), skull fracture and/or cerebral contusion], traumatic subarachnoid hemorrhage (tSAH), midline offset, time from trauma to initial surgery, and intraoperative blood loss and additional DC in the initial operation.

### Prognostic assessment

In all patients, the neurologic outcome was evaluated using the Glasgow Outcome Scale (GOS) at discharge. The definition of GOS score is as follows: 1 indicates death, 2 indicates a persistent vegetative state, 3 indicates severe disability, 4 indicates moderate disability, and 5 indicates good recovery. A GOS score of 1–3 indicates a poor outcome and a GOS score of 4–5 indicates a favorable outcome.

### Statistical analysis

A univariate analysis was performed to assess for risk factors in patients with initial intracranial operation, which predicts an unplanned contralateral second craniotomy. A subsequent unconditional multivariate logistic regression analysis followed. Continuous variables were classified into categorical variables based on clinically applicable cutoff values. All variables were presented in percentage and were compared using the Chisquare test. A p value < 0.05 was considered to have statistical significance. All statistical analysis was performed using SPSS (version 20.0; IBM Corp., Armonk, New York, USA).

## Results

### Baseline characteristics

**Fig. 1:**
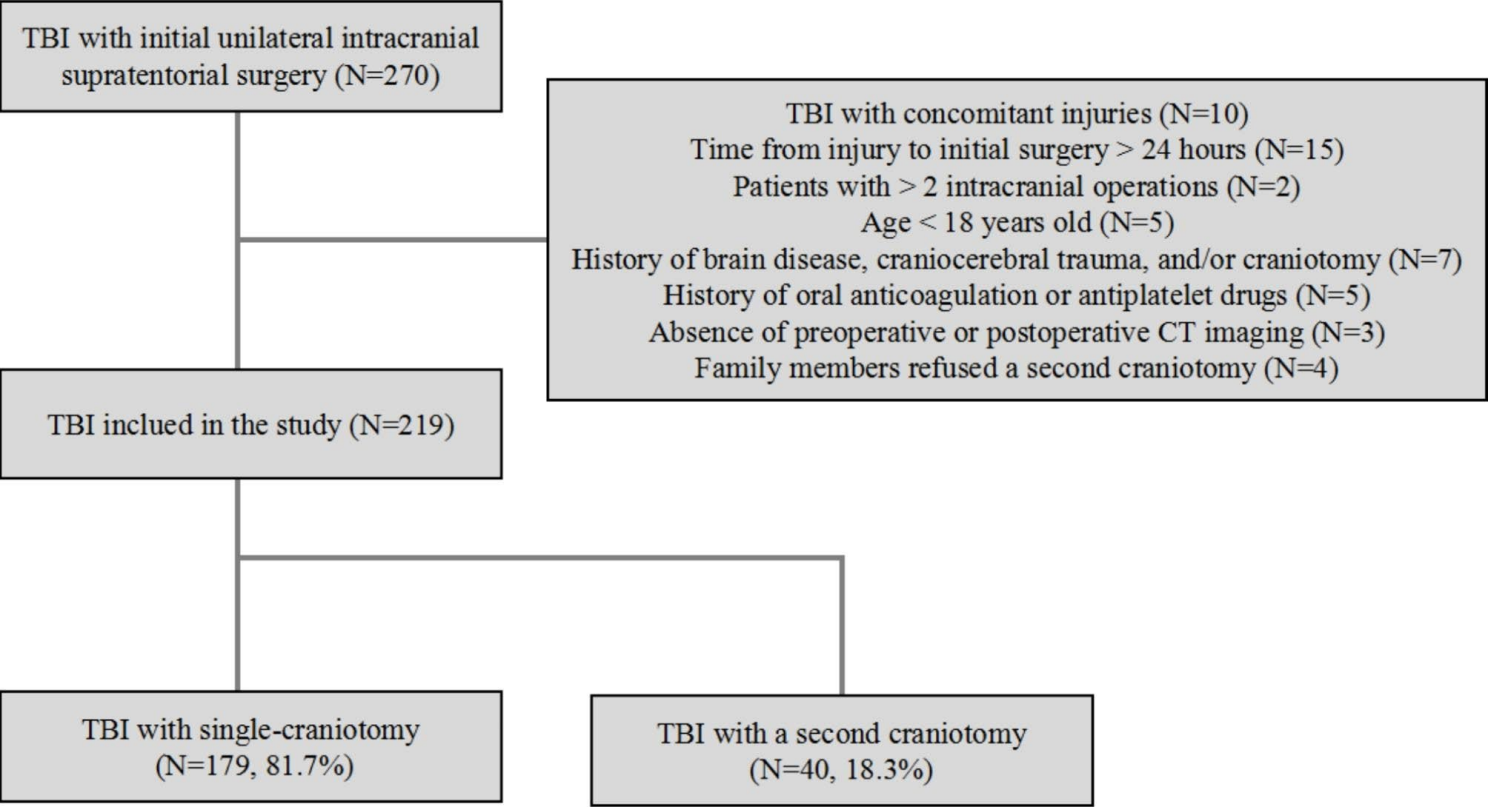
Flowchart of patient screening. *CT* computerized tomography, *TBI* traumatic brain injury

A total of 270 patients with initial unilateral intracranial supratentorial surgery were reviewed in this study. Based on the above inclusion and exclusion criteria, 219 patients were finally included in the analysis, among whom there were 179 patients (81.7%) who underwent intracranial surgery only once, and 40 patients (18.3%) who required an unplanned second craniotomy (Fig. 1). Of the 40 patients with a second craniotomy, eight patients (20%) were age > 60 years old and 34 patients (85%) were male. Traffic accidents were the most common cause of injury (21/40; 52.5%), followed by falls (10/40; 25%), free-falls (7/40; 17.5%) and blows to the head (2/40; 5%). In addition, preoperative brain herniation occurred in 11 patients (27.5%), and traumatic coagulopathy happened in 16 patients (40%). A second craniotomy was performed on the ipsilateral side in 15 patients (37.5%), the contralateral side in 21 patients (52.5%) and the bilateral side in four patients (10%) (Table 1).

**Table 1:** Data of patients who underwent a second craniotomy

Parameters	n (%)
Number of patients	40 (100)
Age, years (> 60)	8 (20)
Male sex	34 (85)
Mechanism of injury	
Traffic accident	21 (52.5)
Free fall	7 (17.5)
Fall	10 (25)
Blow injury	2 (5)
Brain herniation*	11 (27.5)
Traumatic coagulopathy*	16 (40)
Location of second operation	
Ipsilateral side	15 (37.5)
Contralateral side	21 (52.5)
Bilateral side	4 (10)

* pre-operation

### Causes of second craniotomy

**Fig. 2:**
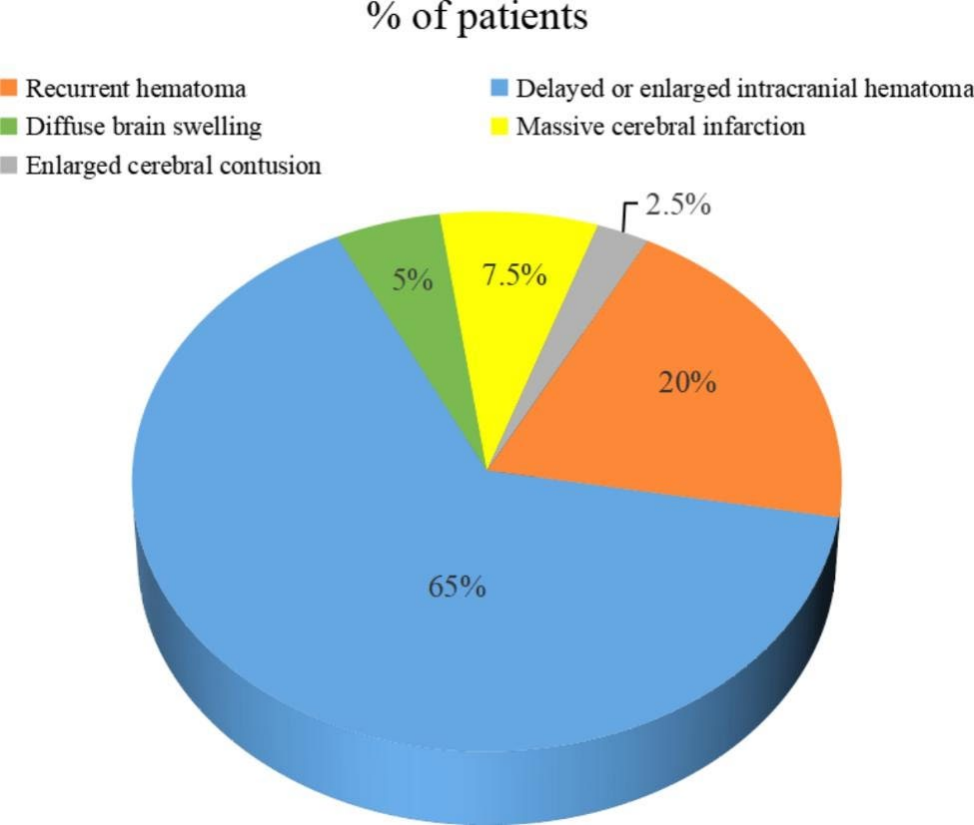
The number of patients with different causes for second craniotomy

**Fig. 3:**
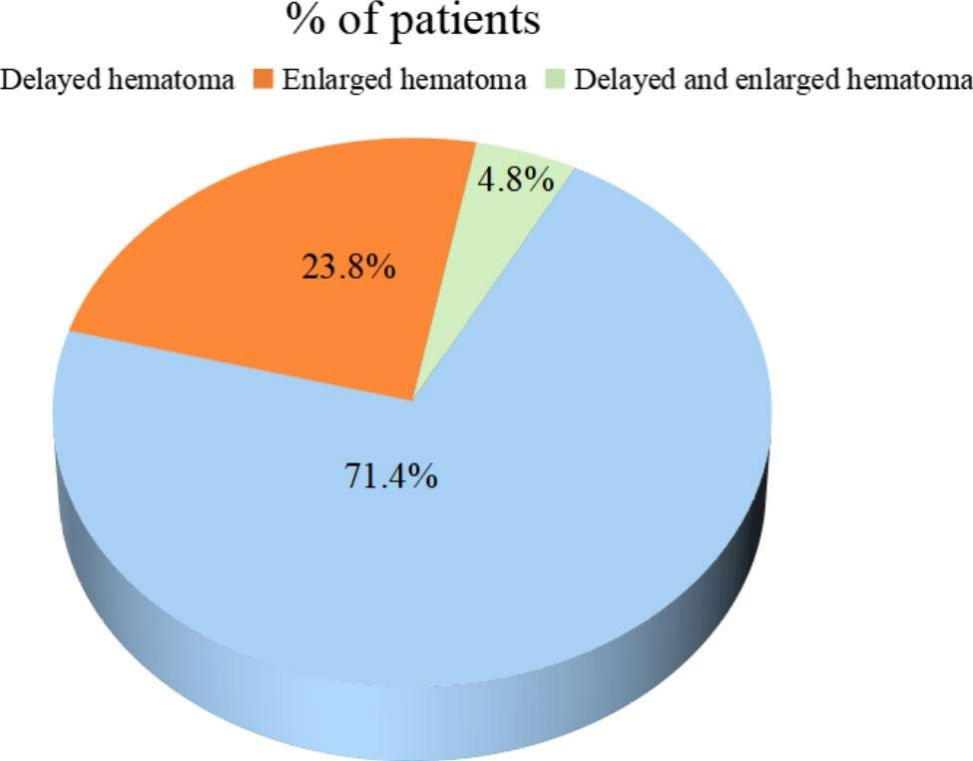
The number of patients with different causes for contralateral second craniotomy

In this study, the initial surgery of 40 patients with a second craniotomy exclusively involved intracranial hematoma and/or cerebral contusion evacuation, among whom there were 20 patients who underwent additional DC. Almost all patients received routine CT scan immediately after initial surgery. If reoperation was necessary, a second craniotomy with hematoma evacuation or DC was performed immediately in patients under the informed consent of the family members. The most common cause for an unplanned second craniotomy was delayed or enlarged hematoma in the non-operation area (26/40; 65%), followed by recurrent hematoma in the operation area (8/40; 20%), ipsilateral massive cerebral infarction (3/40; 7.5%), diffuse brain swelling (2/40; 5%) and enlarged cerebral contusion (1/40; 2.5%) (Fig. 2). Of the 21 patients with a contralateral second craniotomy, 15 (71.4%) experienced contralateral delayed hematoma after initial operation, including seven cases of intracerebral hematoma, six cases of EDH, one case of EDH combined with intracerebral hematoma and one case of SDH. Furthermore, there were 5 (23.8%) patients who experienced contralateral enlarged hematoma after initial operation, including two cases of intracerebral hematoma and three cases of EDH. Another patient (4.8%) experienced simultaneously contralateral enlarged SDH and delayed intracerebral hematoma after initial operation (Fig. 3).

**Fig. 4:**
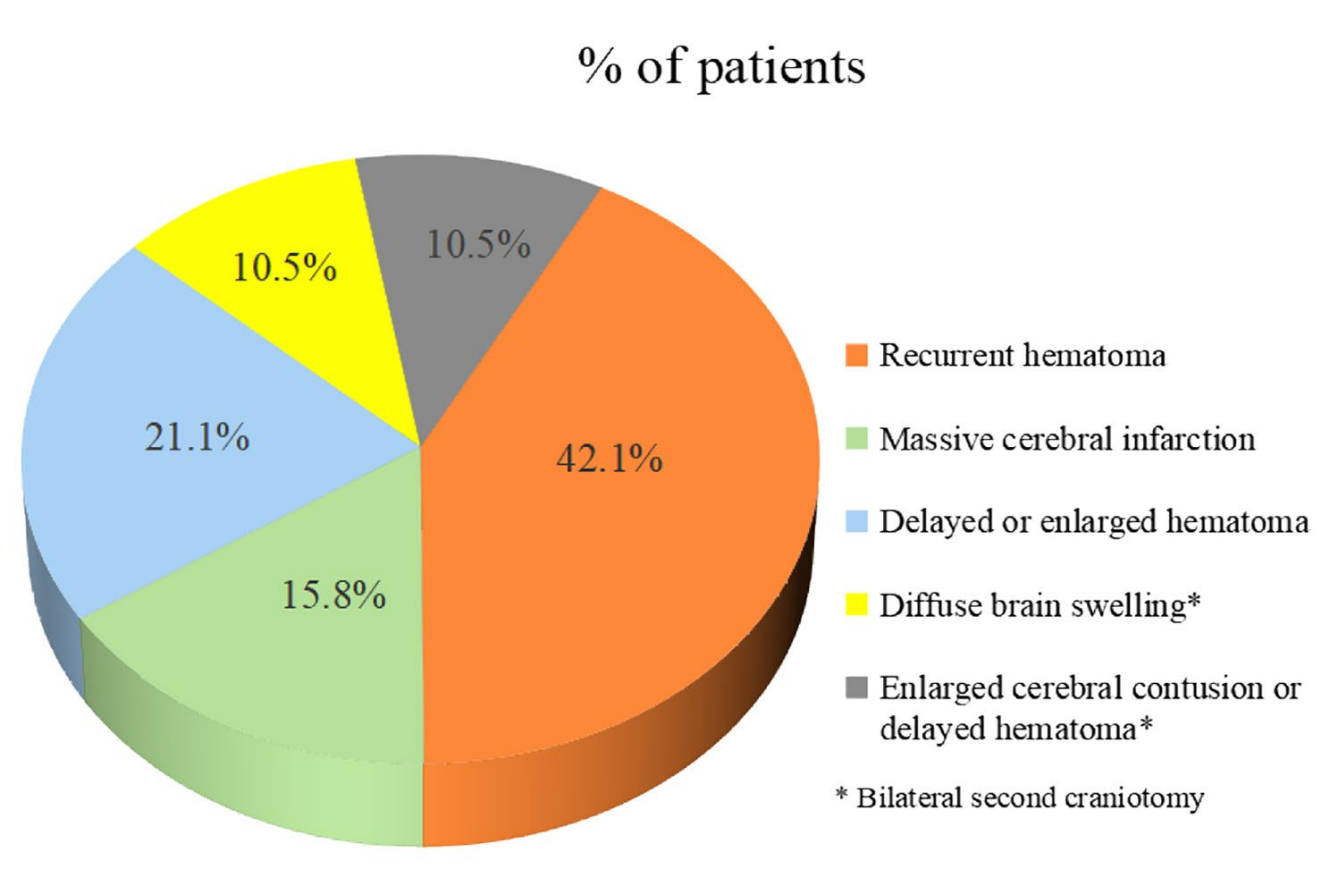
The number of patients with different causes for ipsilateral/bilateral second craniotomy

Of the 19 patients with an ipsilateral/bilateral second craniotomy, 8 (42.1%) experienced recurrent hematoma in the operation area, 3 (15.8%) experienced ipsilateral massive cerebral infarction and 4 (21.1%) experienced delayed or enlarged hematoma in the non-operation area. In addition, a bilateral second craniotomy was performed in two patients with diffuse brain swelling (10.5%) and two patients with enlarged cerebral contusion or delayed hematoma (10.5%) after their initial operation, respectively (Fig. 4). There were two patients who concurrently experienced pore cranial drilling and external ventricular drainage in the treatment of ventricular hemorrhage caused by recurrent or delayed intracerebral hematoma during the second craniotomy. Another patient concurrently experienced an ipsilateral second craniotomy and contralateral pore cranial drilling in the treatment of the chronic subdural effusion after ipsilateral DC in the initial operation.

### Prognosis of second craniotomy

The mean hospital stay of patients with a second craniotomy was 43.8 (range 1–190) days, and the mean GOS score at discharge was 2.3 (range 2–4). Only one patient (2.5%) exhibited a favorable outcome, and the other 39 patients (97.5%) had a poor prognosis. The mean GOS score of patients with a contralateral second craniotomy was 2.4 (range 2–3), which indicates that all patients had a poor prognosis at discharge.

### Risk factors for contralateral second craniotomy

In the univariate analyses, twelve variables contributing to an unplanned contralateral second craniotomy were analysed separately. Consequently, only two factors were found to be signifcantly related to a contralateral second craniotomy (Table 2). The proportion of patients with the CCIF (*P* = 0.001) or additional DC in the initial surgery (*P* = 0.035) were significantly different between the contralateral second craniotomy and ingle-craniotomy. Of the 21 patients with a contralateral second craniotomy, 14 (66.7%) experienced additional DC in the initial operation and 20 (95.2%) had a CCIF on CT image. In contrast, only 76 (42.5%) with additional DC and 102 (57%) with CCIF of the 179 patients who underwent craniotomy only once.

**Table 2:** Univariate analysis of contralateral second craniotomy

Clinical indices	Substratifications	Contralateral second craniotomy (*n* = 21) (%)	Single-craniotomy (*n* = 179) (%)	*P-*value
Sex	male	18 (85.7)	131 (73.2)	0.213
Age, years	18–60	16 (76.2)	133 (74.3)	0.776
	> 60	5 (23.8)	46 (25.7)	
Basic diseases	yes	0 (0)	17 (9.5)	0.288
GCS on admission, scores	3–5	4 (19)	40 (22.3)	0.662
	6–8	10 (47.6)	67 (37.4)	
	> 8	7 (33.3)	72 (40.2)	
tSAH*	yes	19 (90.5)	126 (70.4)	0.051
Midline offset*	yes	18 (85.7)	128 (71.5)	0.165
CCIF*	yes	20 (95.2)	102 (57)	0.001
Brain herniation*	yes	6 (28.6)	56 (31.3)	0.799
Traumatic coagulopathy*	yes	10 (47.6)	54 (30.2)	0.105
Time from injury to surgery†, hours	< 6	14 (66.7)	80 (44.7)	0.064
	6–12	7 (33.3)	70 (39.1)	
	> 12	0 (0)	29 (16.2)	
Intraoperative blood loss†, ml	< 400	10(47.6)	103 (57.5)	0.346
	400–800	8(38.1)	65 (36.3)	
	> 800	3 (14.3)	11 (6.1)	
DC†	yes	14 (66.7)	76 (42.5)	0.035

*CCIF* contralateral craniocerebral injury feature, *DC* decompressive craniectomy, *GCS* Glasgow Coma Scale, *tSAH* traumatic subarachnoid hemorrhage

^*^ pre-operation, ^†^ initial intracranial operation

The multivariate logistic regression analysis showed that CCIF was independent risk factor for a contralateral second craniotomy in patients with initial craniotomy. The odds ratio (OR) and 95% confidence interval (CI) of this variable were 13.175 and 1.715–101.220 in Table 3, respectively. However, no independent association was observed between the proportion of patients with additional DC in the initial operation and a contralateral second craniotomy.

**Table 3:** Multivariate logistic regression analysis of contralateral second craniotomy

Clinical indices	Standard error	OR	95% CI	*P-*value
CCIF (yes)	1.040	13.175	1.715–101.220	0.013
DC (yes)	0.501	2.043	0.766–5.452	0.154

*CCIF* contralateral craniocerebral injury feature, *CI* confidence interval, *DC* decompressive craniectomy, *OR* odds ratio

## Discussion

To the best of our knowledge, this is the first retrospective case-control study to explore the causes and risk factors of an unplanned second craniotomy in patients with TBI. In this study, 53.3% of the 15 patients with an ipsilateral second craniotomy experienced recurrent hematoma in the operation area after initial surgery. Actually, it is quite difficult to rule out the possibility of the incomplete hemostasis caused by operators with different operation experience, which may lead to an unplanned second craniotomy. Therefore, the risk factors for an ipsilateral second craniotomy were not analyzed in this study.

This study retrospectively analyzed 40 patients with a second craniotomy and concluded that the causes of the second craniotomy were as follows: (1) new intracranial hematoma, including recurrent hematoma and delayed hematoma, (2) enlarged cerebral contusion or intracranial hematoma, (3) massive cerebral infarction and (4) diffuse brain swelling. Certainly, the incomplete hemostasisof the operator is associated with the recurrent hematoma in the operation area. In addition, the progression of hemorrhagic injury is also closely related to traumatic coagulopathy in patients with TBI [9]. According to our definition, only one of the eight patients with postoperative recurrent hematoma had traumatic coagulopathy before their initial surgery. However, craniotomy, as an unique trauma to patient’s body, potentially affects the intraoperative coagulation function of the patient with TBI, which may result in new or enlarged intracranial hematoma and even an unplanned second surgery. Among the three patients who experienced ipsilateral massive cerebral infarction after initial surgery, two cases were secondary to the compression of initial or postoperative recurrent extracerebral hematoma, and another patient was secondary to the space-occupying effect of the contralateral subdural effusion after ipsilateral DC. In the absence of iatrogenic damage of blood vessels during operation, the occurrence of postoperative massive cerebral infarction is most likely associated with compressed cerebral surface vessels caused by the extravascular space-occupying lesion [3, 4]. In addition, the use of dehydrating agents increases the viscosity of blood, which may promote the occurrence of cerebral infarction [4]. In this study, 67.5% of the 40 patients with a second craniotomy had delayed or enlarged intracranial hematoma in the non-operation area after initial surgery, one of whom, with cerebral contusion that further expanded into hematoma. The most common mechanisms of delayed or enlarged hematoma are related to the disappearance of the tamponade effect caused by intracranial hypertension, and contralateral skull fracture [8, 10].

Multivariate logistic regression analysis showed that CCIF (OR = 13.175, *P* = 0.013) was independent risk factor for a contralateral second craniotomy. In this study, 95.2% of the 21 patients with a contralateral second craniotomy had CCIF on CT image. There was only one patient without CCIF who underwent a second surgery on the contralateral side, and this case with contralateral delayed SDH experienced the powerful management of intracranial hypertension after initial surgery, which may result in the rebleeding of ruptured blood vessel due to a loss of encephalic pressure. The previous study revealed that initial fracture hematoma (extra-axial haematoma) and contralateral skull fracture features were the two important CT scanning signs, which predicted the occurrence of contralateral EDH after acute SDH evacuation [11]. Actually, the traumatic EDH is associated with injured dural surface vessels caused by skull fracture, and is only one of the important reasons for a contralateral second operation. In this study, 47.6% of the 21 patients with a contralateral second craniotomy experienced contralateral delayed or enlarged EDH after initial surgery, the proportion of which was similar to the patients with contralateral delayed or enlarged intracerebral hematoma. In this study, the CCIF, defined on preoperative CT image including EDH, SDH, intracerebral hematoma, skull fracture and/or cerebral contusion, was a reliable sign that enables the prediction of a contralateral second craniotomy comprehensively. We suggest that CCIF is an adequate yet unnecessary condition for a contralateral second craniotomy. Notably, compared with above previous study that defined contralateral skull features as complex petrous fracture, suture diastasis and fractures involving foramen spinosum or middle meningeal groove, we didn’t provide a detailed definition of skull fracture, which may not specifically predict contralateral delayed or enlarged EDH.

Despite the remedial second surgery were performed timely, 97.5% of 40 patients with a second craniotomy had a poor outcome at discharge in this study. The complete hemostasis of operator could effectively prevent postoperative rebleeding in the operation area. The early evacuation of EDH adjacent to the big cerebral vessels and effective prevention and treatment of subdural effusion after DC could avoid the occurrence of ipsilateral massive cerebral infarction. Lin et al. [12] had established an early-warning scoring model for massive cerebral infarction after acute EDH evacuation, which enabled decision-making regarding additional DC to prevent the occurrence of massive cerebral infarction. Wan et al. [13] suggested that early cranioplasty was the most effective method for preventing or treating contralateral intractable subdural effusion. Some intraoperative phenomenon could predict or identify the contralateral delayed hematoma, including acute brain swelling or malignant intracranial hypertension, massive surgical blood loss, long duration of surgery, craniotomy with large area and pupillary dilation [7, 8, 11, 14]. In addition, the certain techniques of gradient intracranial decompression had been widely used to prevent the presence of the contralateral delayed hematoma, such as the drilling of holes before craniotomy, slow cutting of the duramater, external ventricular drainage combined with gradual decompression and gradual decompression under intracranial pressure monitoring [15–17]. In this study, the slowly cutting of the duramater was performed in most patients with TBI during the initial surgery, and the monitoring of intracranial pressure was performed in a few patients.

However, this study had certain limitations. First, risk factors for an ipsilateral/bilateral second craniotomy were not analyzed. Second, the limited number of patients with a contralateral second craniotomy reduced the statistical power of our study. Third, the definition of traumatic coagulopathy lacks a unified standard, which might have influenced our statistical results.

## Conclusion

An unplanned second craniotomy was relatively common among patients with TBI, and their outcomes were often poor. All these efforts with causes analysis and risk factor evaluation aim to prevent an unplanned second craniotomy and improve patients’ outcomes. It also can help neurosurgeons to take more accurate and prompt treatment measures. This study evaluated the clinical data of 40 patients with TBI who underwent a second craniotomy. The second craniotomy can be implemented on the ipsilateral, contralateral or bilateral side due to new intracranial hematoma, enlarged cerebral contusion or intracranial hematoma, massive cerebral infarction or diffuse brain swelling. A contralateral second craniotomy was often caused by delayed or enlarged intracranial hematoma, and multivariate logistic regression analysis showed that its high-risk factor was CCIF defined on preoperative CT scanning. Above all, early postoperative reexamination of cranial CT is helpful for timely detection of above catastrophic events and remedial measures.

## Data Availability

The datasets used and/or analysed during the current study available from the corresponding author on reasonable request.

## Abbreviations

CCIF: Contralateral craniocerebral injury feature
CT: computerized tomography
DC: Decompressive craniectomy
EDH: Extradural hematoma
GCS: Glasgow Coma Scale
GOS: Glasgow Outcome Scale
SDH: Subdural hematoma
TBI: Traumatic brain injury
tSAH: Traumatic subarachnoid hemorrhage
